# In Vivo Evaluation of Bulk Metallic Glasses for Osteosynthesis Devices

**DOI:** 10.3390/ma9080676

**Published:** 2016-08-09

**Authors:** Kazuhiro Imai, Sachiko Hiromoto

**Affiliations:** 1 Department of Life Sciences, Graduate School of Arts and Sciences, The University of Tokyo, Tokyo 153-8902, Japan; 2 National Institute for Materials Science, Tsukuba, Ibaraki 305-0047, Japan; hiromoto.sachiko@nims.go.jp

**Keywords:** bulk metallic glasses, in vivo evaluation, osteosynthesis devices

## Abstract

Bulk metallic glasses (BMGs) show higher strength and lower Young’s modulus than Ti-6Al-4V alloy and SUS 316L stainless steel. This study aimed to perform in vivo evaluations of Zr_65_Al_7.5_Ni_10_Cu_17.5_ BMGs for osteosynthesis devices. In the study for intramedullary implants, osteotomies of the femoral bones were performed in male Wistar rats and were stabilized with Zr_65_Al_7.5_Ni_10_Cu_17.5_ BMGs, Ti-6Al-4V alloy, or 316L stainless steel intramedullary nails for 12 weeks. In the study for bone surface implants, Zr_65_Al_7.5_Ni_10_Cu_17.5_ BMGs ribbons were implanted on the femur surface for 6 weeks. Local effects on the surrounding soft tissues of the implanted BMGs were assessed by histological observation. Implanted materials’ surfaces were examined using scanning electron microscopy equipped with energy dispersive X-ray spectroscopy (SEM-EDS). In the study for intramedullary implants, bone healing after osteotomy was assessed by peripheral quantitative computed tomography (QCT) and mechanical tests. Histological observation showed no findings of the biological effects. SEM-EDS showed no noticeable change on the surface of BMGs, while Ca and P deposition was seen on the Ti-6Al-4V alloy surface, and irregularities were seen on the 316L stainless steel surface. Mechanical test and peripheral QCT showed that, although there was no significant difference, bone healing of BMGs was more than that of Ti-6Al-4V alloy. The results indicated that Zr-based BMGs can lead to bone healing equal to or greater than Ti-6Al-4V alloy. Zr-based BMGs exhibited the advantage of less bone bonding and easier implant removal compared with Ti-6Al-4V alloy. In conclusion, Zr-based BMGs are promising for osteosynthesis devices that are eventually removed.

## 1. Introduction

Many biomaterials have been developed and used for medical and dental device materials. Osteosynthesis devices such as intramedullary nails, bone plates, and screws are widely used for bone fracture management. Metallic materials including Ti-6Al-4V alloy and SUS 316L stainless steel are widely used for osteosynthesis devices because load-bearing is required, and metals have high mechanical strength. Those metallic materials used for osteosynthesis devices need to have high strength, low Young’s modulus, and good biocompatibility. They also have the material characteristics of durability, innocuity, and corrosion resistance. However, current osteosynthesis devices sometimes fail or break by corrosion fatigue and fretting corrosion fatigue due to their insufficient strength and durability [1]. To prevent failure, currently-available bone plates are thick and bulky. Extensive surgical exposure is necessary to implant such thick and bulky plates, and it is sometimes difficult to close the wound after implantation. Regarding intramedullary nails, larger implant diameter is necessary to prevent nail failure. Such larger nails occupy most of the medullary canal and interfere with the blood supply, and may lead to bone healing disturbance. One of the problems with the current metallic materials is their insufficient strength. Another problem is their excessively high Young’s modulus. Currently available metallic devices have Young’s moduli of around 100 GPa—much higher than that of cortical bone (20 GPa). This sometimes causes stress shielding and absorption of the bone stabilized with osteosynthesis devices made of these materials [2,3,4,5,6].

Bulk metallic glasses (BMGs), also known as amorphous metals, are solid metallic materials with a disordered atomic-scale structure. Most metals are crystalline in their solid state, with a highly-ordered arrangement of atoms. Crystalline alloys have grain boundaries, dislocations, segregations, and slip planes. Due to grain boundaries, dislocations, and segregations, they can easily undergo failure against loading. Slip planes of crystalline alloys are moved by shear stress, following plastic deformation. BMGs are non-crystalline and have glass-like structures. BMGs have random and disordered atomic structures and do not have any segregations, defects, or slip planes. When BMGs are loaded, they transform by movement of the mass of atoms, and the elastic transformation continues until the applied load reaches their yield load. Therefore, BMGs have the mechanical properties of higher strength and lower Young’s modulus. The tensile strength of the Zr-based BMGs is much higher than that of Ti-6Al-4V alloy and 316L stainless steel. The Young’s modulus of the Zr-based BMGs is closer to that of bone than Ti-6Al-4V alloy and 316L stainless steel [7]. Osteosynthesis devices made of BMGs have higher strength and lower Young’s modulus than currently available metallic devices, and might solve the problems listed above.

Three basic rules for the formation of BMGs have been reported [8]. BMGs are formed as alloys rather than pure metal. The alloy has to be made of at least three elements. The atomic radii of the elements have to be significantly different. The atomic size differences should be more than 12% among the main constituent elements. Additionally, the combination of components should have negative heat of mixing, inhibiting crystal nucleation, and prolonging the time that the molten metal stays in a supercooled state. The BMGs contain atoms of significantly different sizes, leading to low free volume in the molten state. The viscosity prevents the atoms from moving enough to form an ordered lattice. By choosing appropriate compositions which satisfy the three basic rules, BMGs based on Mg, Ca, lanthanide metal (Ln), Ti, Zr, Hf, Fe, Co, Ni, Pd, Pt, Cu, and Au were produced by various solidification processes. To use BMGs as osteosynthesis devices, large thickness or diameter formation is essential. BMGs with large diameters of >20 mm are produced based on Mg, Ln, Zr, Fe, Ni, Pd, Pt, and Cu [9,10,11,12,13,14,15,16].

There have been limited in vivo experiments of BMGs. Fe-based BMG (Fe-Cr-Si-B) ribbons and Co-based BMG (Co-Fe-Si-B, Co-Fe-Nb-Si-B, Co-Si-B) ribbons implanted in the femoral muscles of mature rabbits showed the deposition of Fe, Cr, and Si elements in tissues surrounding the ribbons, and marked corrosion was recognized. In addition, a marked pathological inflammation was noticed around the Co-based BMG ribbons, and the implanted ribbons had completely lost their original shape after 6-weeks of implantation [17]. After this negative result, no in vivo experiments of BMGs had been reported for more than two decades. Instead of Fe-based or Co-based BMGs, Zr-based BMGs (Zr_65_Al_7.5_Ni_10_Cu_17.5_) showed excellent anti-corrosiveness in simulated body fluid [18,19]. In addition, Zr_52.5_Cu_17.9_Ni_14.6_Al_10.0_Ti_5.0_ BMGs showed excellent electrochemical properties in the phosphate-buffered saline electrolyte [20].

Corrosion behaviors of the Zr-based BMGs were evaluated in phosphate buffered solution by electrochemical polarization, and the results showed that the Zr-based BMGs exhibited excellent corrosion resistance [21,22,23]. The corrosion resistance of the Zr-based BMGs can be attributed to the formation of passive films on the surface of alloys. Chemical compositions for the surface of the Zr-based BMGs were studied by X-ray photoelectron spectroscopy (XPS). The results showed that the oxide film formed on the surface of the Zr-based BMGs consisted of ZrO_2_, Al_2_O_3_, and Nb_2_O_5_ [22,23]. The results indicated that the enriched oxides provide Zr-based BMGs with high corrosion resistance and prevent corrosive attack in phosphate buffered solution. The potential cytotoxicity of the BMGs was evaluated by 1 week cell culture, and the results indicated that they had biocompatibility as good as Ti-6Al-4V [21]. Likewise, normal cell adhesions and cell morphologies after 4-h incubation suggested the initial biosafety and biocompatibility of Zr-based BMGs [22]. The behaviors of BMGs studied in vivo have not been investigated so far.

The aim of this article is to cover the recent studies of in vivo evaluations of Zr-based Zr_65_Al_7.5_Ni_10_Cu_17.5_ BMGs for osteosynthesis devices [24,25]. In the study for intramedullary implants, osteotomies of the femoral bones were performed in male Wistar rats and were stabilized with Zr_65_Al_7.5_Ni_10_Cu_17.5_ BMGs, Ti-6Al-4V alloy, or 316L stainless steel intramedullary nails for 12 weeks. Systemic effects and local effects were assessed by measuring Cu and Ni concentrations in the blood and surrounding soft tissue of the implanted BMGs. The surfaces of the BMGs after implantation were examined using scanning electron microscopy equipped with energy dispersive X-ray spectroscopy (SEM-EDS). Bone healing after femur osteotomy was assessed by peripheral quantitative computed tomography (pQCT) and mechanical test. In the study of bone surface implants, Zr_65_Al_7.5_Ni_10_Cu_17.5_ BMGs were implanted sub-periosteally on the femoral bone surface for 6 weeks. Local effects of the surrounding soft tissues were assessed by histological observation and the surfaces of the BMGs were examined by SEM-EDS. These studies were performed to evaluate the potential of BMGs as biomaterials for osteosynthesis devices.

## 2. Materials and Methods

### 2.1. Preparation of Materials

Pure metals—each with a purity of more than 99.9%—consisting of zirconium, aluminum, nickel, and copper were mixed, and a Zr_65_Al_7.5_Ni_10_Cu_17.5_ alloy ingot was prepared. The alloy ingot was prepared by an arc melting method. The ingot was crushed into small pieces, packed into a quartz nozzle, and re-melted using a high-frequency induction furnace in a vacuum. The melted alloy in the nozzle was ejected into a copper mold with 2.0 mm diameter and 40 mm length and rapidly supercooled. The alloy was cut into 35 mm length. Accordingly, Zr_65_Al_7.5_Ni_10_Cu_17.5_ BMG nails with 2.0 mm diameter and 35 mm length were obtained. Next, re-melted alloys were rapidly supercooled by casting on the rotating copper roll, and Zr_65_Al_7.5_Ni_10_Cu_17.5_ BMGs ribbons with 10 mm length, 2 mm width, and 0.5 mm thickness were obtained. The formed alloy structure was analyzed using X-ray diffraction (XRD) with Cu Kα radiation to confirm that the alloy structure was amorphous, verifying that the prepared materials are BMGs. XRD patterns and transmission electron microscope (TEM) images of Zr_65_Al_7.5_Ni_10_Cu_17.5_ BMG nails, in addition to XRD patterns of Zr_65_Al_7.5_Ni_10_Cu_17.5_ BMG ribbons were reported previously [18,26]. The XRD patterns showed that the structure of the prepared alloys were amorphous.

The surfaces of BMG nails and ribbons were polished with 600 grit silicon carbide paper. The BMG materials were dried to generate passive film, and before implantation surgery, they were sterilized in a high-pressure BS-305 steam sterilizer (TOMY Co., Tokyo, Japan). Ti-6Al-4V alloy K-wires with 2.0 mm diameter (Mathys Medical Ltd., Bettlach, Switzerland) and 316L stainless steel K-wires with 2.0 mm diameter (Mathys Medical Ltd., Bettlach, Switzerland) were prepared as the compared materials. The K-wires were cut into 35 mm length, and before implantation surgery, they were sterilized in a high-pressure steam sterilizer. Mechanical properties of the prepared materials are summarized in Table 1.

### 2.2. Surgical Procedure

The in vivo experiment was approved by the animal experimentation committee based on in vitro studies which showed excellent corrosion resistance in simulated body fluid [18,19]. Male Wistar rats—14-week-old and weighing 380 to 490 grams—were used for all experiments. The animals were housed in a separate cage with a size of 22 cm × 33 cm × 13 cm, and received a standard diet. 

Thirty-five animals were randomly assigned to four groups. In 14 animals, Zr_65_Al_7.5_Ni_10_Cu_17.5_ BMG nails were inserted intramedullary of the femoral bone (BMG nail group). In the other 14 animals, Ti-6Al-4V K-wires were implanted intramedullary (Ti-6Al-4V group). 316L stainless steel K-wires were implanted intramedullary in four animals (316L group). Zr_65_Al_7.5_Ni_10_Cu_17.5_ BMG ribbons were implanted sub-periosteally on the femoral bone surface in three animals (BMG ribbon group). Surgical procedure was performed under 75 mg/kg ketamine hydrochloride and 10 mg/kg xylazine anesthesia.

**Figure 1:**
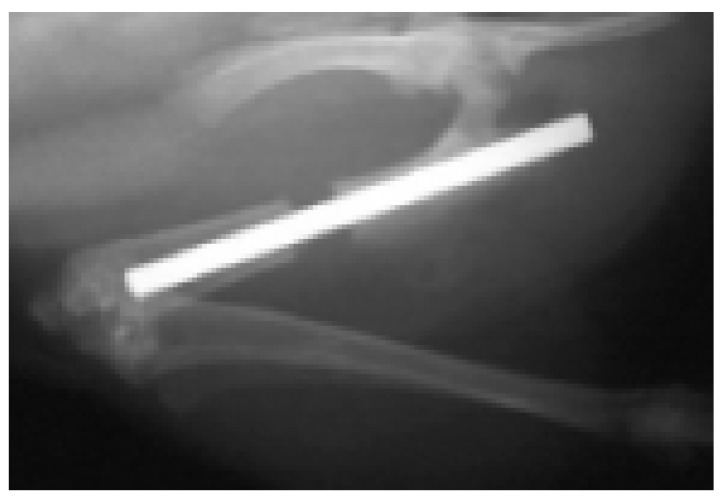
Radiograph of left femoral bone just after osteotomy and BMG intramedullary nail implantation.

In the BMG nail group, the Ti-6Al-4V group, and the 316L group, osteotomy of left femoral bone was performed at 13 mm from the top of the greater trochanter using a fine-toothed circular saw. The medullary canal of the femoral bone was reamed using 1.5 mm and 2.0 mm diameter drill bits (Mathys Medical Ltd., Bettlach, Switzerland). After the reaming procedure, the nail was inserted from the trochanter to the femoral condyle, and the disconnected femoral bone was manually reduced (Figure 1). The contra-lateral right femoral bone in the BMG nail group, the Ti-6Al-4V group, and the 316L group was left intact.

**Figure 2:**
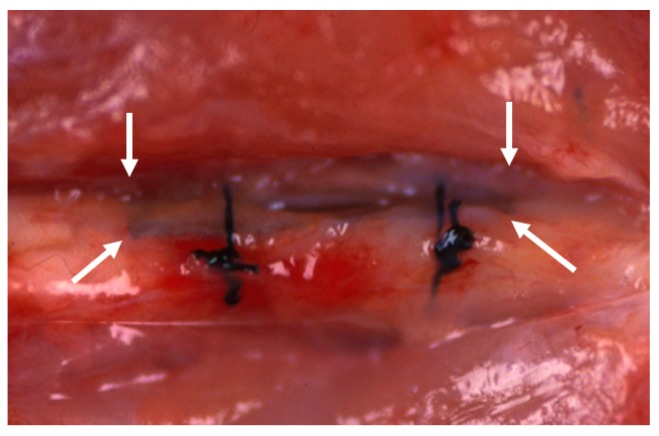
BMG ribbon (arrows) was implanted sub-periosteally of the left femoral bone. BMG ribbon was tied up to the femoral bone on both the proximal side and the distal side with 4-0 nylon suture string to prevent its movement.

In the BMG ribbon group, the BMG ribbon was implanted sub-periosteally of the left femoral bone of each animal. The BMG ribbon was tied to the femoral bone on both the proximal side and the distal side with 4-0 nylon suture string to prevent its movement (Figure 2). A sham operation was performed on the right femoral bone in the BMG ribbon group. 

After the implantation, the animals were allowed to move freely in their own cage. Duration of implantation was 12 weeks in the BMG nail group, the Ti-6Al-4V group, and the 316L group; and 6 weeks in the BMG ribbon group.

### 2.3. Evaluation of Systemic and Local Effects

At the end of intramedullary nail implantation, blood concentrations of Cu and Ni were measured to assess systemic effects. Under anesthesia with intra-peritoneal injection of 45 mg/kg ketamine hydrochloride and 6 mg/kg xylazine, 5 mL of blood was obtained from the descending aorta. Then, euthanasia was carried out with 120 mg/kg ketamine hydrochloride and 16 mg/kg xylazine intra-peritoneal injection. The periosteum and gluteal muscles surrounding the implanted nail were excised. The amounts of Cu and Ni contained in the excised tissues were measured using graphite furnace atomic absorption spectroscopy Z-8100 (Hitachi Ltd., Tokyo, Japan). In the BMG ribbon group, undecalcified specimens of the femoral bone, periosteum, and surrounding soft tissues in contact with the BMG ribbon were made, following Hematoxylin–Eosin staining procedure.

### 2.4. Evaluation of Implant Surface

After the implantation, the implanted nails and ribbons were removed under euthanasia. Left femoral bones in the BMG nail and Ti-6Al-4V group were harvested and preserved by freezing at −70 °C. The removed implants were cleaned with detergent in purified water for 20 s and stored in airtight cases. The surfaces of the implants were examined by SEM XL30FEG (Philips, Eindhoven, The Netherlands). Deposited substances on the surfaces of the implants were identified by quantitative elemental analysis with energy dispersion X-ray spectroscopy (EDS) DX-4 (EDAX Japan, Tokyo, Japan).

### 2.5. Evaluation of Bone Healing after Osteotomy

In the BMG nail and Ti-6Al-4V groups, bone healing was quantitatively assessed by pQCT and mechanical test after 12 weeks of implantation. The harvested femoral bones were scanned with pQCT system XCT Research SA + (Stratec Medizintechnik GmbH, Pforzheim, Germany) with a voxel size of 0.08 × 0.08 × 0.5 mm^3^. Three consecutive cross-sections with 0.2 mm inter-slice distance were obtained at each osteotomy site. The mean value of the three sections was used for evaluation. Cortical bone was defined by threshold value of 690 mg/cm^3^. The bone mineral density of cortical bone (CoBMD, mg/cm^3^), the bone mineral content of cortical bone (CoBMC, mg/mm), the cross-sectional area of cortical bone (CoCSA, mm^2^), and the stress–strain index (SSI) were measured.

**Figure 3:**
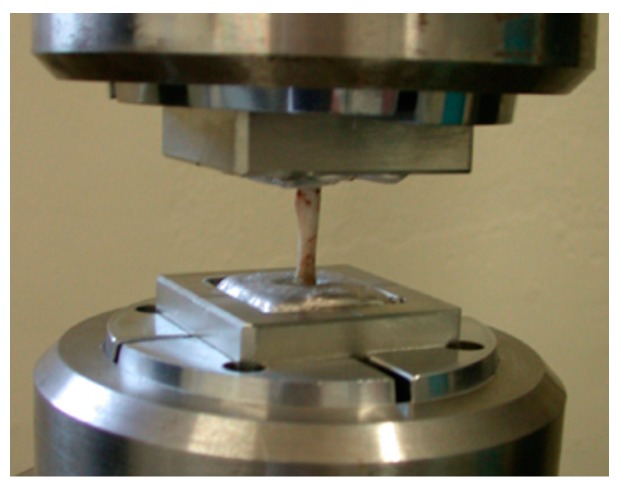
Torsional mechanical test of the harvested femoral bone.

Just prior to the mechanical test, the femoral bones were unfrozen at room temperature. Both ends of the measured bone were fixed with Wood’s metal U-70 (Asahi Metal, Osaka, Japan) to keep the bone axis alignment during the torsional mechanical test. Torsion test at 15 degrees/minute angular velocity was performed using a torsional mechanical test machine STTM-A (T.S. Engineering, Kanagawa, Japan) without any axial loading (Figure 3). The measured maximum torque was defined as the ultimate torque during the torsional test. Torsional stiffness was defined as the maximum torque ratio to the rotational angle when the ultimate torque was reached. Energy absorption corresponding to the energy required for bone failure was calculated from the torque—angular displacement curve. Measured values from the BMG nail group were compared with those from the Ti-6Al-4V group using the unpaired Student’s *t* test. Differences were considered significant at *p* < 0.05.

## 3. Results

### 3.1. Systemic and Local Levels of Cu and Ni

In the evaluation of the systemic effects of the BMG nail group, the mean whole blood Cu concentration and Ni concentration were 147.8 ± 43.2 (mean ± standard deviation) μg/dL and less than 0.10 μg/dL, respectively. In the Ti-6Al-4V group, the mean whole blood Cu concentration and Ni concentration were 153.1 ± 38.3 μg/dL and 0.11 ± 0.03 μg/dL, respectively. In the 316L group, the mean whole blood Cu concentration and Ni concentrations were 145.8 ± 13.6 μg/dL and less than 0.10 μg/dL, respectively. There were no differences between Cu and Ni levels in the BMG nail group compared to those of the Ti-6Al-4V group and the 316L group (Table 2).

The local Cu and Ni levels in the surrounding tissues of the BMG nail group were 10.2 ± 12.6 μg/g and 2.9 ± 2.4 μg/g, respectively. In the Ti-6Al-4V group, the Cu and Ni levels were 22.3 ± 42.6 μg/g and 2.3 ± 1.0 μg/g, respectively. In the 316L group, the Cu and Ni levels were 10.4 ± 8.0 μg/g and less than 2.0 μg/g, respectively. The results showed no difference between Cu or Ni in the surrounding soft tissues of the BMG nail group compared with the Ti-6Al-4V group or the 316L group.

### 3.2. Histological Findings

**Figure 4:**
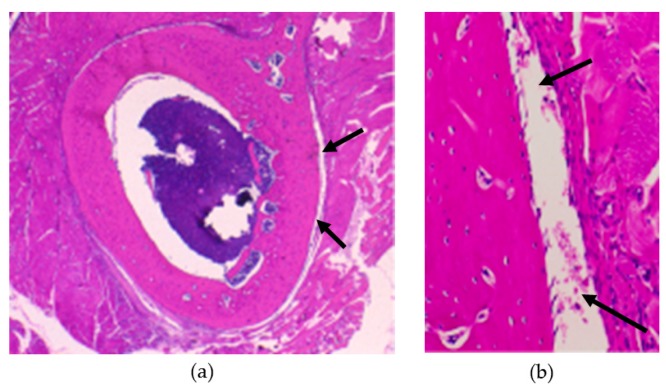
Histological pictures of the femoral bone and the surrounding soft tissues after 6 weeks of implantation and removal of BMG ribbon. BMG ribbon had been implanted sub-periosteally (arrows). (**a**) ×10; (**b**) ×100.

H&E-stained specimens of the femoral bone and the surrounding soft tissues in contact with the BMG ribbon were observed using light microscopy. There was no observation of biological effects such as bone resorption, infiltration of inflammatory cells, cell necrosis, cellular dysplasia, or wear debris (Figure 4).

### 3.3. Evaluation of Implant Surface

**Figure 5:**
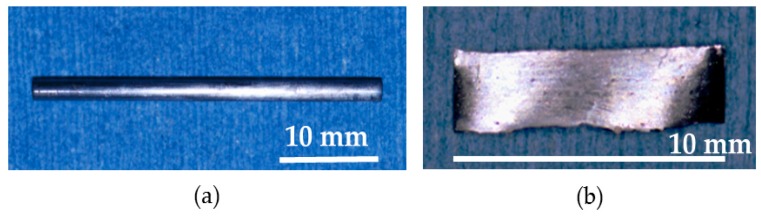
Removed (**a**) BMG nail and (**b**) BMG ribbon. There was no failure, no breakage, and no pitting corrosion.

**Figure 6:**
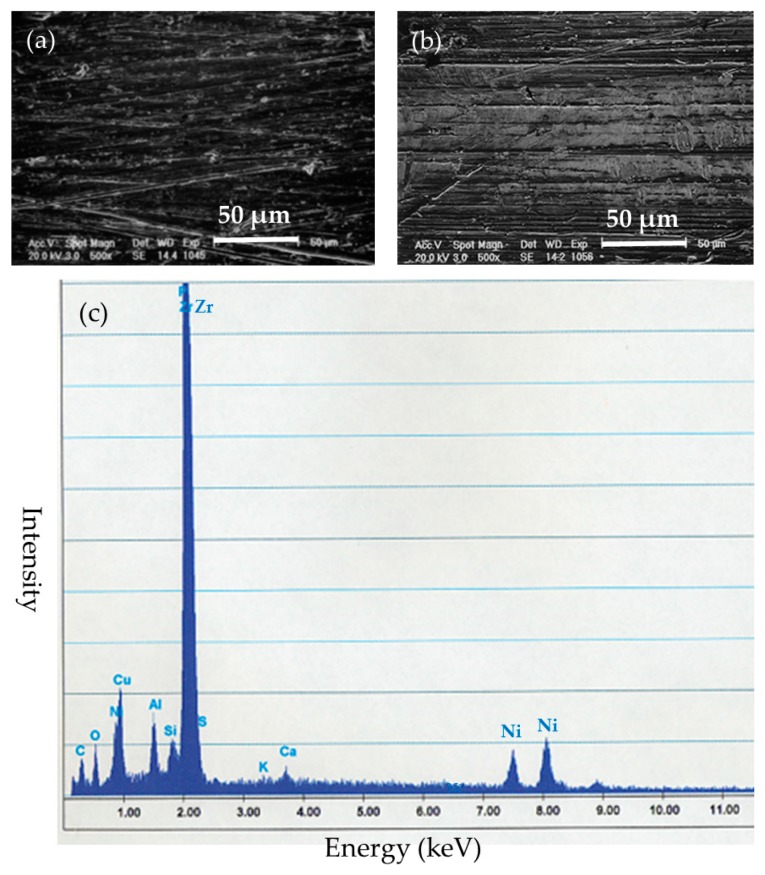
(**a**) Surface SEM image of the intraosseous part of the BMG nail before implantation; (**b**) Surface SEM image of the intraosseous part of the BMG nail after 12 weeks of implantation. There was no difference between before and after implantation; (**c**) scanning electron microscopy equipped with energy dispersive X-ray spectroscopy (SEM-EDS) evaluation of the intraosseous part of the BMG nail.

**Figure 7:**
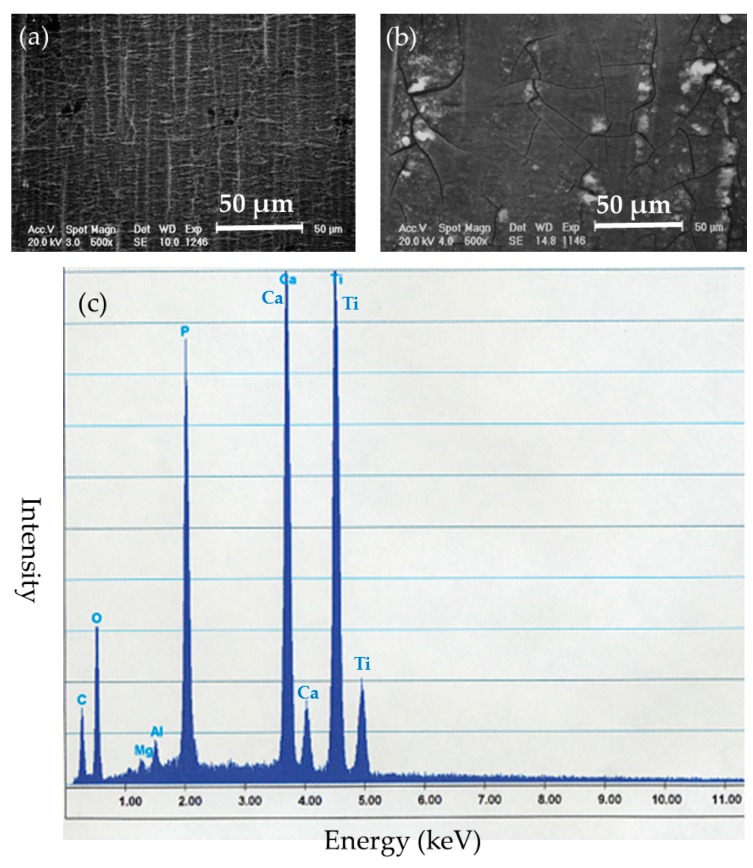
(**a**) Surface SEM image of the intraosseous part of the Ti-6Al-4V alloy nail before implantation; (**b**) Surface SEM image of the intraosseous part of the Ti-6Al-4V alloy nail after 12 weeks of implantation; (**c**) SEM-EDS evaluation of the intraosseous part of the Ti-6Al-4V alloy nail. Substantial amounts of deposits with Ca and P were identified.

**Figure 8:**
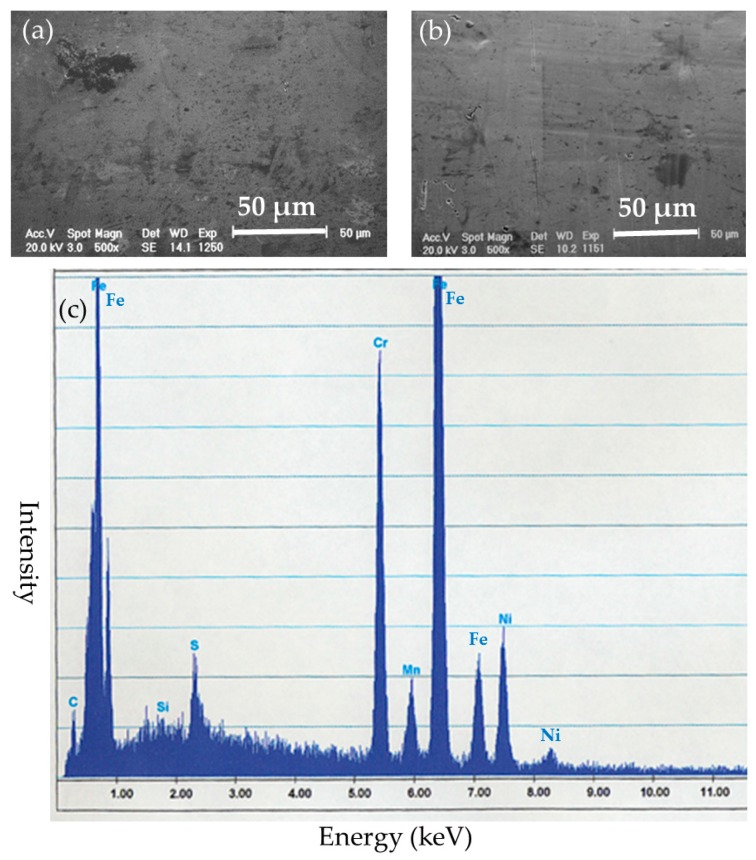
(**a**) Surface SEM image of the intraosseous part of the 316L stainless steel nail before implantation; (**b**) Surface SEM image of the intraosseous part of the 316L stainless steel nail after 12 weeks of implantation; (**c**) SEM-EDS evaluation of the intraosseous part of the 316L stainless steel nail. Surface irregularities and micro-pits were observed, and a large amount of sulfur deposition was identified.

Surface evaluation of the BMG implants was performed to assess corrosion resistance and durability. There was no failure, no breakage, and no pitting corrosion of the removed BMG nails and ribbons (Figure 5). Observation by SEM showed transverse scars made by the polishing procedure. There was no difference in surface morphology between before and after implantation in SEM-EDS observation (Figure 6a,b). Surface compositions of the removed implants were semi-quantitatively evaluated using SEM-EDS. In BMG nails, a slight amount of Ca and substantial amount of P were identified by SEM-EDS at the site of the nail in the femoral bone (Figure 6c). On Ti-6Al-4V alloy nails, laminated deposits were observed by SEM, and substantial amounts of Ca and P were identified by SEM-EDS at the intraosseous parts after 12 weeks of implantation (Figure 7). On 316L nails, surface irregularities and micro-pits probably formed by the retrieval procedure with thin corrosion product layers observed by SEM, and a large amount of sulfur deposition was identified by SEM-EDS after 12 weeks of implantation (Figure 8).

**Figure 9:**
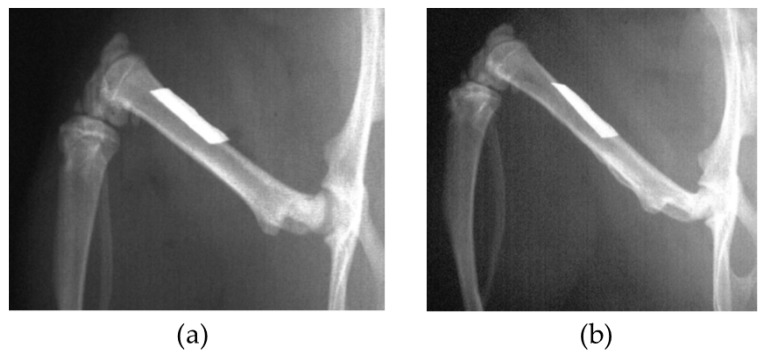
(**a**) Radiograph of left femoral bone just after BMG ribbon implantation; (**b**) Radiograph of femoral bone and BMG ribbon at 6 weeks after implantation. The size and shape of the ribbon have not changed from the original ones.

Regarding BMG ribbons, radiograph examinations with CMB-2 (Softex, Kanagawa, Japan) showed that there was no difference between just after and at 6 weeks post-implantation. The size and shape of the ribbons did not change. In addition, there was no morphological change in the femoral bone (Figure 9).

### 3.4. Evaluation of Bone Healing

**Figure 10:**
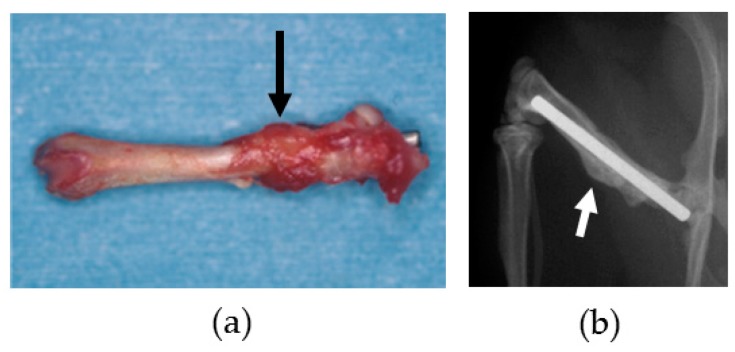
(**a**) Harvested femoral bone with BMG nail; (**b**) Radiograph of femoral bone and BMG nail at 12 weeks after osteotomy. The osteotomy sites were healed with callus formation (arrow).

The osteotomy sites were healed with callus formation after 12 weeks of nail implantation (Figure 10). The CoBMD of the osteotomy site was 1036 ± 74 mg/cm^3^ in the BMG nail group, and 1046 ± 70 mg/cm^3^ in the Ti-6Al-4V group. There was no significant difference between the two groups (*p* = 0.87). The CoBMC of the BMG nail and the Ti-6Al-4V groups were 14.4 ± 3.7 mg/mm and 12.0 ± 5.8 mg/mm, respectively. The difference between the two groups was not significant (*p* = 0.37). The CoCSA in the BMG nail and the Ti-6Al-4V groups were 13.8 ± 3.0 mm^2^ and 11.7 ± 5.9 mm^2^, respectively. The difference was not significant (*p* = 0.40). The SSI values in the BMG nail and the Ti-6Al-4V group were 21.5 ± 5.6 and 15.7 ± 9.6, respectively. The difference also failed to reach significance (*p* = 0.19), but the SSI in the BMG nail group was greater than that in the Ti-6Al-4V group.

**Figure 11:**
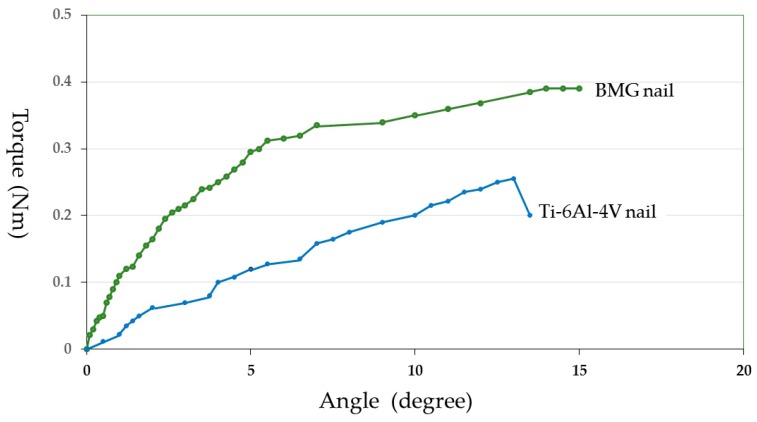
Torque angle curve of the harvested femoral bone at 12 weeks after osteotomy and BMG nail implantation and Ti-6Al-4V nail implantation.

The maximum torque in the BMG nail group and the Ti-6Al-4V groups were 0.34 ± 0.19 Nm and 0.18 ± 0.16 Nm, respectively. The difference was not significant (*p* = 0.14), but higher strength was attained in the BMG nail group compared with the Ti-6Al-4V group. The torsional stiffness was 0.044 ± 0.035 Nm/degree in the BMG nail group and 0.020 ± 0.021 Nm/degree in the Ti-6Al-4V group. The difference was not significant (*p* = 0.19), although the torsional stiffness in the BMG nail group was higher than that in the Ti-6Al-4V group. The energy absorption in the BMG nail and the Ti-6Al-4V groups were 2.2 ± 2.2 Nm degree and 1.0 ± 0.9 Nm degree, respectively. The value in the BMG nail group was greater than that in the Ti-6Al-4V group, but the difference was not significant (*p* = 0.27). The representative torque angle curve of the BMG nail group showed torsional yield and higher maximum torque, indicating that the bone healing after osteotomy had almost been completed (Figure 11). In contrast, the torque angle curve of the Ti-6Al-4V group showed fracture without torsional yield with lower maximum torque, indicating that the bone healing had not yet been completed (Figure 11).

## 4. Discussion

From the results of the Cu and Ni levels in the blood and the surrounding soft tissue, the histological observation, and the radiograph examination, there were no systemic effects and local effects after Zr_65_Al_7.5_Ni_10_Cu_17.5_ BMG implantation. 

The SEM-EDS results indicated that Zr-based BMGs did not derive apparent deposition of calcium phosphate, which generally causes strong adhesion to bone in vivo, but showed a zirconium phosphate layer, which does not show bone adhesion. Ti-6Al-4V alloy was liable to form calcium phosphate deposits on its surface. Titanium and its alloys show excellent biocompatibility which leads to osseous integration and strong bone-to-metal attachment. 316L stainless steel tended to suffer from general corrosion, with the preferential adsorption of proteins containing –SH groups.

The detaching test after Ti-6Al-4V implantation indicated that the titanium alloy bonds directly to bone after more than 8 weeks [27]. Because of the stronger bonding to the bone, a higher rate of complications than with stainless steel pins occurred during the removal of titanium pins in patients with slipped capital femoral epiphysis [28]. In addition, a retrospective review showed that removal of the titanium intramedullary nails of the femoral bone required a significantly longer surgery time than removal of the stainless steel nails [29]. Therefore, osteosynthesis devices with low reactivity (such as Zr-based BMGs) might be more appropriate in situations with a future implant removal. Recently, Ti-based BMG (Ti_40_Zr_10_Cu_34_Pd_1__4_Sn_2_) bars were implanted in the femoral bone of rats, and local tissue reaction as well as their component ions’ diffusion in local area and whole body were evaluated. The in vivo study showed that Ti-based BMGs have good tissue compatibility, equivalent bone integration, and bonding ability [30]. Ti-based BMGs might be more appropriate in the situation without a future removal.

Regarding osteotomy healing, the Zr-based BMG nail experiment did not show any significant differences compared to the Ti-6Al-4V alloy assessed by pQCT and torsional mechanical test. However, the mean value of each parameter was larger for the BMG group than that for Ti-6Al-4V group. Zr-based BMG intramedullary nails promoted bone healing equal to or greater than Ti-6Al-4V alloy.

Concerning BMG ribbons, a previous in vivo experiment reported that Co-based BMG ribbons caused a marked pathological inflammation around the ribbons, and the implanted ribbons completely lost their original shape after a 6-week implantation [17]. In contrast, this in vivo experiment using Zr-based BMGs showed that there was no biological effect in the surrounding tissues, and no failure, no breakage, and no pitting corrosion in the removed ribbons. The differences in the results between Co-based BMG ribbons and Zr-based BMG ribbons is assumed to be due to the materials used. Zr-based BMGs are high strength, have good corrosion resistance, and good biocompatibility.

There are problems in the utilization of Zr-based BMGs for clinical application as osteosynthesis devices at this moment. One of the problems is that even larger-sized BMG materials should be manufactured for osteosynthesis devices, which is technically demanding to create. Another problem is that there is no data about long term BMG implantation with durability, non-toxicity, and corrosion resistance.

The size of BMGs is limited depending on its glass-forming ability [31]. Balancing BMG mechanical properties and size may be required, because free volume content (which governs the mechanical property) depends on the fabrication processes. Recently, studies of thin film metallic glasses (TFMGs) have been performed. TFMGs have an extrinsic size effect on hardness and fracture mechanisms [32]. The change of the mechanical properties of TFMGs might come from a change of atomic arrangement and free volume content. TFMGs have unique properties, and suggest the possibility of taking greater advantage of metallic glasses and their enhanced mechanical properties [33]. Beneficial mechanical properties, such as high corrosion resistance and wear resistance of TFMGs, have been reported. Therefore, TFMGs have potential as a high corrosion and wear-resistant coating material for biomaterials. Furthermore, combination of BMGs and TFMGs may enhance the application of metallic glasses. TFMGs of Ti_40_Cu_36_Pd_14_Zr_10_ covering 316L stainless steel were investigated in vitro and showed high corrosion resistance [34].

## 5. Conclusions

This in vivo study showed that Zr-based BMG intramedullary nails promoted bone healing equal to or greater than Ti-6Al-4V alloy. In addition, SEM-EDS results showed that Zr-based BMGs were inactive in vivo. Zr-based BMGs exhibited the advantage of less bone bonding and easier implant removal compared with Ti-6Al-4V alloy.

Osteosynthesis devices with low reactivity—such as Zr-based BMGs—are assumed to be more appropriate in situations with a future implant removal, such as intramedullary nails, bone plates, and screws. BMGs are a promising metallic biomaterial for new osteosynthesis devices, but further manufacturing technique and in vivo study are necessary. In conclusion, Zr-based BMGs are promising for osteosynthesis devices that are eventually removed.

## Figures and Tables

**Table 1:** Mechanical properties of Zr_65_Al_7.5_Ni_10_Cu_17.5_ bulk metallic glasses (BMGs), Ti-6Al-4V alloy, and 316L stainless steel, compared to human bone.

Materials	Ultimate Tensile Strength (MPa)	Young’s Modulus (GPa)
Zr_65_Al_7.5_Ni_10_Cu_17.5_ BMGs	1500–1700	70–80
Ti-6Al-4V alloy	950	108–116
316L stainless steel	>480	203
Human bone (femur)	120	15–20

**Table 2:** Whole blood Cu and Ni concentrations in the BMG nail group, the Ti-6Al-4V group, and the 316L group at 12 weeks post-implantation.

Materials	BMG Nail Group (*n* = 14)	Ti-6Al-4V Group (*n* = 14)	316L Group (*n* = 4)	Normal Value
Cu (μg/dL)	147.8 ± 43.2	153.1 ± 38.3	145.8 ± 13.6	80–150
Ni (μg/dL)	<0.10	0.11 ± 0.03	<0.10	<0.60
